# Collagenolytic and gelatinolytic matrix metalloproteinases and their inhibitors in basal cell carcinoma of skin: comparison with normal skin

**DOI:** 10.1054/bjoc.1999.0978

**Published:** 2000-01-18

**Authors:** J Varani, Y Hattori, Y Chi, T Schmidt, P Perone, M E Zeigler, D J Fader, T M Johnson

**Affiliations:** Departments of Pathology and Dermatology, The University of Michigan Medical School, 1301 Catherine Road, PO Box 0602, Ann Arbor, 48109, USA

**Keywords:** interstitial collagenase, collagenase-3, tissue inhibitoral metalloproteinase invasion, fibroblast, epithelial cells, endothelial cells

## Abstract

Tissue from 54 histologically-identified basal cell carcinomas of the skin was obtained at surgery and assayed using a combination of functional and immunochemical procedures for matrix metalloproteinases (MMPs) with collagenolytic activity and for MMPs with gelatinolytic activity. Collagenolytic enzymes included MMP-1 (interstitial collagenase), MMP-8 (neutrophil collagenase) and MMP-13 (collagenase-3). Gelatinolytic enzymes included MMP-2 (72-kDa gelatinase A/type IV collagenase) and MMP-9 (92-kDa gelatinase B/type IV collagenase). Inhibitors of MMP activity including tissue inhibitor of metalloproteinases-1 and -2 (TIMP-1 and TIMP-2) were also assessed. All three collagenases and both gelatinases were detected immunochemically. MMP-1 appeared to be responsible for most of the functional collagenolytic activity while gelatinolytic activity reflected both MMP-2 and MMP-9. MMP inhibitor activity was also present, and appeared, based on immunochemical procedures, to reflect the presence of TIMP-1 but not TIMP-2. As a group, tumours identified as having aggressive-growth histologic patterns were not distinguishable from basal cell carcinomas with less aggressive-growth histologic patterns. In normal skin, the same MMPs were detected by immunochemical means. However, only low to undetectable levels of collagenolytic and gelatinolytic activities were present. In contrast, MMP inhibitor activity was comparable to that seen in tumour tissue. In previous studies we have shown that exposure of normal skin to epidermal growth factor in organ culture induces MMP up-regulation and activation. This treatment concomitantly induces stromal invasion by the epithelium (Varani et al (1995) *Am J Pathol***146**: 210–217; Zeigler et al (1996 *b*) *Invasion Metastasis***16**: 11–18). Taken together with these previous data, the present findings allow us to conclude that the same profile of MMP/MMP inhibitors that is associated with stromal invasion in the organ culture model is expressed endogenously in basal cell carcinomas of skin. © 2000 Cancer Research Campaign


*British Journal of Cancer* (2000) **82**(3), 657–665
© 2000 Cancer Research Campaign
DOI: 10.1054/ bjoc.1999.0978, available online at http://www.idealibrary.com on IDEAL®

### Collagenolytic and gelatinolytic matrix metalloproteinases and their inhibitors in basal cell carcinoma of skin: comparison with normal skin

**J Varani[1], Y Hattori[1], Y Chi[1], T Schmidt[1], P Perone[1], ME Zeigler[1], DJ Fader[2] and TM Johnson[2]**

Departments of [1]Pathology and [2]Dermatology, The University of Michigan Medical School, 1301 Catherine Road, PO Box 0602, Ann Arbor, MI 48109, USA

**Summary** Tissue from 54 histologically-identified basal cell carcinomas of the skin was obtained at surgery and assayed using a combination of functional and immunochemical procedures for matrix metalloproteinases (MMPs) with collagenolytic activity and for MMPs with gelatinolytic activity. Collagenolytic enzymes included MMP-1 (interstitial collagenase), MMP-8 (neutrophil collagenase) and MMP-13 (collagenase-3). Gelatinolytic enzymes included MMP-2 (72-kDa gelatinase A/type IV collagenase) and MMP-9 (92-kDa gelatinase B/type IV collagenase). Inhibitors of MMP activity including tissue inhibitor of metalloproteinases-1 and -2 (TIMP-1 and TIMP-2) were also assessed. All three collagenases and both gelatinases were detected immunochemically. MMP-1 appeared to be responsible for most of the functional collagenolytic activity while gelatinolytic activity reflected both MMP-2 and MMP-9. MMP inhibitor activity was also present, and appeared, based on immunochemical procedures, to reflect the presence of TIMP-1 but not TIMP-2. As a group, tumours identified as having aggressive-growth histologic patterns were not distinguishable from basal cell carcinomas with less aggressive-growth histologic patterns. In normal skin, the same MMPs were detected by immunochemical means. However, only low to undetectable levels of collagenolytic and gelatinolytic activities were present. In contrast, MMP inhibitor activity was comparable to that seen in tumour tissue. In previous studies we have shown that exposure of normal skin to epidermal growth factor in organ culture induces MMP up-regulation and activation. This treatment concomitantly induces stromal invasion by the epithelium (Varani et al (1995) *Am J Pathol* **146**: 210–217; Zeigler et al (1996*b*) *Invasion Metastasis* **16**: 11–18). Taken together with these previous data, the present findings allow us to conclude that the same profile of MMP/MMP inhibitors that is associated with stromal invasion in the organ culture model is expressed endogenously in basal cell carcinomas of skin. © 2000 Cancer Research Campaign

**Keywords:** interstitial collagenase; collagenase-3; tissue inhibitoral metalloproteinase invasion; fibroblast; epithelial cells; endothelial cells

Invasion into the adjacent stroma is the initial event in the process by which epithelial tumours spread to distant sites. A number of experimental approaches have been developed to study the invasion process (Kramer and Nicolson, 1979; Mareel et al, 1979; Erkell and Schirrmacher, 1988; Reich et al, 1988; Huber et al, 1992; Varani et al, 1997; Rosenthal et al, 1998). While useful for studying invasion in experimental tumours, these approaches are not amenable for the study of human tumours under in situ conditions. Recently we described a human skin organ culture model that can be used to investigate mechanisms of tissue invasion in situ. This model consists of small pieces of normal human skin maintained in organ culture under serum-free conditions. In the absence of exogenous growth factors, normal histological features and biochemical functions are preserved (Varani et al, 1993*a*, 1993*b*, 1994). However, when an exogenous source of growth factors is included in the culture medium, the epithelium becomes hyper-proliferative and invasion of the underlying stroma by epithelial cells occurs (Fligiel and Varani, 1993; Zeigler et al, 1996*a*). Since invasion in this model is initiated by addition of exogenous growth factors to the culture medium, the cellular and molecular changes that occur concomitantly with invasion can be identified. Furthermore, we can use this model to interfere with cellular and molecular events that occur concomitantly with invasion to directly assess their role in the invasion process.

Matrix metalloproteinases (MMPs) are a family of genetically-related enzymes that play a role in normal tissue development, remodelling and repair (Sympson et al, 1994; Inoue et al, 1995). These same enymes are also responsible for tissue destruction in a number of pathological conditions including acute and chronic inflammation (Mulligan et al, 1993; Galis et al, 1994; Gibbs et al, 1999), ultraviolet light-induced skin damage (Fisher et al, 1996, 1997), rheumatoid arthritis (Hasty et al, 1990) and tumour invasion (Coussens and Werb, 1996). Stromal invasion by growth factor-stimulated epithelial cells in organ-cultured skin appears to be dependent, at least in part, on MMP activity. Acquisition of invasive potential is accompanied by increased synthesis and activation of a number of MMPs (Varani et al, 1995; Zeigler et al, 1996*b*); and invasion is suppressed by addition of exogenous MMP inhibitor to the culture medium (Zeigler et al, 1996*b*).

While this model of stromal invasion allows the pathophysiological events that support invasion to be probed under in situ conditions, it is not clear how closely events in this model mimic those that bring about invasion by actual human epithelial tumours. In order to begin addressing this issue, we have in the present study examined basal cell carcinomas of skin for production of MMPs and MMP inhibitors. Basal cell carcinomas were

*Received 24 March 1999*
*Revised 19 August 1999*
*Accepted 23 August 1999*

*Correspondence to:* J Varani chosen for this study because while metastasis formation is a late event in this tumour, local invasion with extensive tissue destruction is prevalent (Goslen and Bauer, 1986). We report here that levels of collagenolytic and gelatinolytic enzymes present in the tumour tissue (in the absence of exogenous growth factors) are much higher than levels present in normal skin maintained under the same serum-free, growth factor-free conditions, but similar to the levels seen in normal skin following exposure to invasion-promoting growth factors. In contrast, there appear to be no differences in MMP inhibitor levels between tumour tissue and normal tissue maintained under either growth factor-free or growth factor-containing conditions.

### MATERIALS AND METHODS

### Antibodies and reagents

A rabbit polyclonal IgG antibody to MMP-1 was obtained from Chemicon (Temecula, CA, USA). Monoclonal antibodies to MMP-2, MMP-8, MMP-9 and MMP-13 were obtained from Oncogene Sciences (Cambridge, MA, USA). Rabbit IgG antibodies to tissue inhibitor of metalloproteinase-1 (TIMP-1) and TIMP-2 were acquired from Triple Point Biologics (Forest Grove, OR, USA). A mouse monoclonal IgG1 antibody and a rabbit polyclonal IgG antibody were used as controls (Accurate Scientific and Chemical Company, Westbury, NY, USA). Human recombinant TIMP-1, obtained from Oncogene Sciences, and human recombinant TIMP-2, obtained as a generous gift from Dr Keith Langley (Amgen Corp., Thousand Oaks, CA, USA), were also used as controls.

### Basal cell carcinomas and normal skin

Surgically-removed basal cell carcinomas were obtained from 54 different individuals. Ten were from lesions diagnosed as aggressive-growth histological pattern (e.g. morpheaform, infiltrative, sclerosing and micronodular) basal cell carcinoma; with the remaining specimens classified as superficial, nodular or mixed (non-aggressive-growth) types. Upon arrival in the laboratory, a small piece of tissue from each specimen was immediately fixed in 10% buffered formalin and used for histology/immunohistology. Another small piece of tissue was frozen in liquid nitrogen. An extract of the frozen tissue was prepared as described previously for normal skin (Fisher et al, 1996). The remaining tissue was trimmed away from the surrounding normal epidermis as much as possible, cut into 2 × 2-mm, full-thickness pieces (i.e. with the adjacent stroma), and placed in wells of a 24-well dish. Normally 4–6 tissue pieces were placed in 0.5 ml of culture medium. Keratinocyte basal medium (KBM), obtained from Clonetics Inc (Walkersville, MD, USA) was used as culture medium. This serum-free, growth factor-free modification of MCDB-153 medium was supplemented with 1.4 mM $Ca^{2+}$ (final concentration), since previous studies have shown that a physiological $Ca^{2+}$ concentration is required for maintenance of tissue viability (Varani et al, 1993*a*, 1993*b*, 1994). The tissue pieces were incubated for 3 days at 37°C and 5% carbon dioxide/95% air. At the end of the incubation period, culture fluids were collected and used for MMP and MMP inhibitor measurements as described below. The tissue was fixed in 10% buffered formalin and used to assess structural integrity after staining with haematoxylin and eosin.

Sixteen neonatal foreskin specimens obtained at circumcision were used as controls. The normal tissue was fixed in 10% buffered formalin or maintained under the same serum-free, growth factor-free conditions as used with the tumour tissue. The preparation and characteristics of normal skin in organ culture have been described in past reports (Varani et al, 1993*a*, 1993*b*, 1994). Human dermal fibroblasts and human dermal microvascular endothelial cells were also used as controls in certain experiments. Cells were prepared from foreskin tissue as described previously (Varani et al, 1994; Zeigler et al, 1996*a*). Fibroblasts were grown in Dulbecco's modified minimal essential medium of Eagle supplemented with 10% fetal bovine serum (Gibco, Grand Island, NY, USA). The endothelial cells were grown in endothelial growth medium (Clonetics). For use as controls in MMP assays, the fibroblasts were incubated for 3 days in $Ca^{2+}$-supplemented (serum-free) KBM and the endothelial cells were maintained in serum-free endothelial basal medium (Clonetics). In other experiments, human peripheral blood neutrophils, prepared by dextran sedimentation and Ficoll-Hypaque centrifugation (Varani et al, 1985), were used as a control. Neutrophils were suspended in Hanks' balanced salt solution at $1 \times 10^7$ cells $ml^{-1}$ and stimulated for 90 min with 100 nM phorbol myristate acetate. After centrifugation to remove cells, the supernatant (e.g. the releasate), was used as a source of enzymes (MMP-8 and MMP-9) (Grams et al, 1995; Shapiro et al, 1995).

### Collagenase assays

A combination of approaches was used to assess collagenase expression and activity. Collagenase activity in organ culture fluids was quantified as the production of trichloroacetic acid (TCA)-soluble fragments from $^3$H-labelled type I fibrillar collagen (18 h assay) as described previously (Hu et al, 1978). Activity was assessed in the absence of aminophenyl mercuric acetate (APMA) and following exposure to 1 mM APMA for 90 min to activate latent enzyme (Springman et al, 1990). *Clostridium histolyticum* collagenase, obtained from Worthington Biochemicals (Freehold, NJ, USA), was used as a positive control in each experiment. A standard curve was generated in each assay using the bacterial collagenase, and the activity in organ culture fluids was compared to the standard curve. In certain experiments, the same experimental samples were assayed on different days. Variability from day to day was minimal.

As a way to visualize collagen fragmentation, a 1 mg $ml^{-1}$ solution of native type I fibrillar collagen was exposed to control or APMA-treated culture fluids for 18 h. At the end of the incubation period, the collagen samples were resolved on an 8.5% sodium dodecyl sulphate polyacrylamide gel (SDS-PAGE) along with intact collagen that had not been exposed to the culture fluids and with culture fluids without substrate. Activity was indicated by the disappearance of the parent collagen bands and the concomitant appearance of lower molecular weight fragments (Mulligan et al, 1993).

In parallel, organ culture fluids were assessed for ability to digest casein by substrate zymography and examined by Western blotting for reactivity with anti-MMP-1, anti-MMP-8 and anti-MMP-13 antibodies. Mammalian collagenases have the capacity to digest casein (Sottrup-Jensen and Birkedal-Hansen, 1989). Zymography allows for the identification of the molecular size of active fractions. Used in conjunction with Western blotting, it

 © 2000 Cancer Research Campaign provides a means for distinguishing MMP-1 (interstitial collagenase), MMP-8 (neutrophil collagenase) and MMP-13 (collagenase-3). Quantification of latent and active forms was done by laser scanning densitometry. For Western blotting, culture fluid proteins were resolved by SDS-PAGE and transferred to nitrocellulose filters. Following treatment of filters with specific antibodies, the filters were reacted with horseradish peroxidase (HRP)-conjugated secondary antibodies. Reactive proteins were detected by enhanced chemiluminescence (ECL; Amersham) after treatment with liminol and visualization on light-sensitive autoradiography film (Fisher et al, 1996).

Finally, tissue specimens fixed in 10% buffered formalin immediately after surgery were examined by immunoperoxidase staining for MMP-1, MMP-8 and MMP-13 expression (Fisher et al, 1997). Briefly, paraffin-embedded sections were mounted on glass slides coated with poly-L-lysine and stained using the ABC method (Vector Labs; Burlingame, CA, USA). Diaminobenzidine was used as the chromogenic substrate, and tissues were counterstained with haematoxylin. Tissues stained with rabbit or mouse IgGs in place of specific antibodies were used as controls.

### Gelatinase assays

Gelatin zymography was used for the detection of enzymes with gelatinolytic activity including MMP-2 ($M_r$ 72-kDa gelatinase A/type IV collagenase) and MMP-9 ($M_r$ 92-kDa gelatinase B/type IV collagenase). Zymography was performed as described previously (Varani et al, 1995; Zeigler et al, 1996*b*). In parallel, tissue specimens fixed in 10% buffered formalin immediately after surgery were examined by immunoperoxidase staining for MMP-2 and MMP-9 expression.

### MMP inhibitor assays

MMP inhibitor expression was assessed using a combination of functional and immunochemical approaches. To determine levels of functional inhibitor activity, 3-day culture fluids were obtained from basal cell carcinomas or normal skin and treated overnight with 10 mM EDTA to inactivate endogenous MMPs. Following dialysis in four changes of $Ca^{2+}$-free, 0.05 M Tris buffer (pH 7.2), the EDTA-treated and dialysed culture fluids were examined for suppression of human neutrophil MMP activity in a spectrofluometric assay as described in a recent report (Chi et al, 1999). Briefly, human peripheral blood neutrophils were prepared and 'activated' as described above. The neutrophil releasate (containing MMP-8 and MMP-9 but no inhibitor [Shapiro et al, 1995]) was mixed with varying amounts of organ culture fluid and a series of inhibition slopes generated. TIMP-1 was used in parallel to generate a standard curve, and inhibition values obtained with culture fluids were expressed in relation to this. In conjunction with the functional assay, organ culture fluids were examined by Western blotting with antibodies to TIMP-1 and TIMP-2.

### RESULTS

### Collagenase activity in basal cell carcinomas and normal skin

In the first series of experiments, 3-day culture fluids from 18 basal cell carcinomas were assayed for degradation of $^{3}$H-collagen

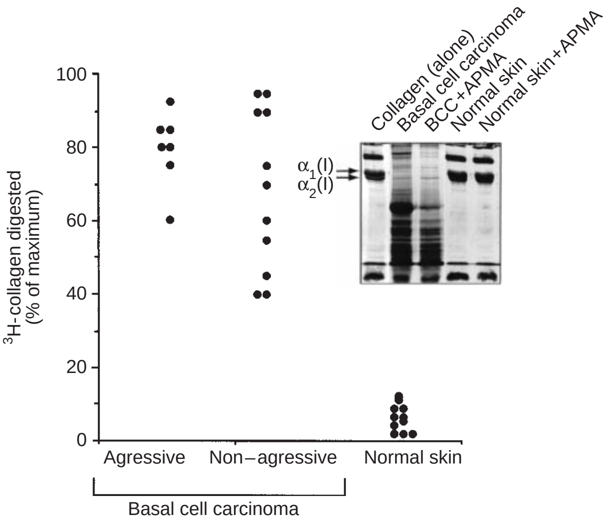

**Figure 1** Dot plot comparing collagenase activity in culture fluids from aggressive growth pattern basal cell carcinomas, from non-aggressive growth pattern tumours and from normal skin. Each dot is representative of an individual tumour or normal tissue specimen. A standard curve was generated using *Clostridium histolyticum* collagenase with each experiment, and culture fluid values determined directly from the standard curve. Experimental values are presented as a percentage of the maximal radioactivity released using the highest concentration of bacterial collagenase (20 ng). Insert: Rat tail collagen was exposed to control and APMA-activated culture fluids from a basal cell carcinoma and a normal skin sample for 18 h as described in the Materials and Methods section. Following this, the treated collagen specimens along with untreated collagen were separated by SDS-PAGE and stained with Coomassie brilliant blue. Intact $\alpha_1$(I) and $\alpha_2$(I) chains (arrows) can be seen in the untreated collagen sample. Decreased intensity of the parent bands and presence of lower molecular weight fragments are indicative of collagen digestion into TCA soluble fragments (Figure 1). High levels of activity were detected in all of the culture fluids. While there was variability from specimen to specimen, the aggressive-growth histological pattern tumours were not different as a group from basal cell carcinomas with non-aggressive histological patterns. Maximal enzymatic activity was observed in the absence of APMA treatment and incubation of the culture fluids with APMA did not further increase the activity (not shown). This indicates that a significant fraction of the MMP enzymes responsible for collagenase activity was present in active rather than latent form. The failure of APMA treatment to increase activity is consistent with the fact that continued fragmentation of MMPs occurs during incubation, and that biological activity can be lost as smaller fragments are generated (Shapiro et al, 1995). Additional studies (not shown) demonstrated that collagenase activity was lost in the presence of 10 mM EDTA but was unaffected by 2 mM phenylmethyl sulphonylfluoride (PMSF). In contrast to what was seen with the tumour culture fluids, there was little activity in 3-day organ culture fluids from normal skin, either in the absence or presence of APMA ($n$ = 12) (Figure 1).

### Characterization of collagenolytic enzymes by casein zymography and Western blotting

Culture fluids from 14 basal cell carcinomas were next examined by casein zymography. An additional 12 specimens of normal skin were examined in parallel. Culture fluids from all of the tumour specimens hydrolysed the casein substrate, with zones of hydrolysis evident at 54 and 45 kDa (consistent with the presence

© 2000 Cancer Research Campaign

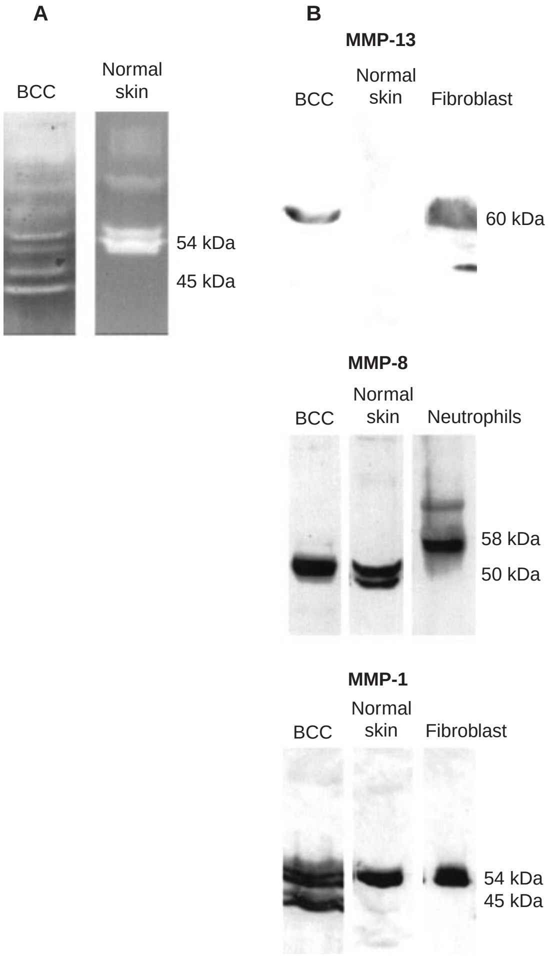

**Figure 2** (**A**) Zymograms demonstrating caseinolytic activity in culture fluids from a representative basal cell carcinoma specimen and from a normal skin specimen. (**B**) Western blots of the same specimens with anti-MMP-13, anti-MMP-8 and anti-MMP-1 antibodies. Human skin fibroblasts and human peripheral blood neutrophils were used as controls

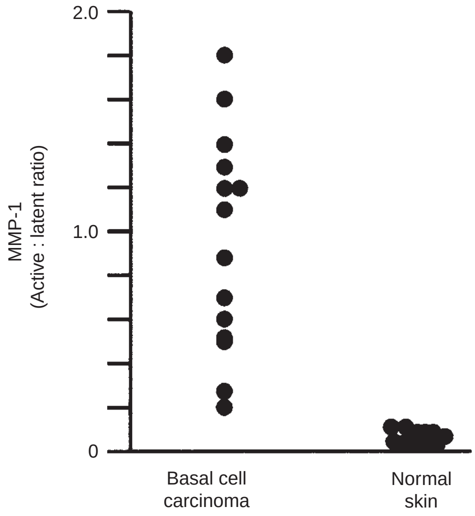

**Figure 3** Dot plot comparing ratios of active to latent MMP-1 bands based on densitometry scanning of casein zymograms. Each dot is representative of an individual tumour or normal tissue specimen of latent and active forms of MMP-1). The casein hydrolytic zones co-migrated identically with zones obtained with culture fluid from control human diploid fibroblasts (54 kDa) and APMA-activated human diploid fibroblasts (54 and 45 kDa). Most specimens demonstrated no activity in the higher molecular weight regions of the gel. In the few specimens demonstrating activity in the 60–70 kDa region of the gel (consistent with the presence of MMP-8 and/or MMP-13), the zones were very faint. In contrast to these results, culture fluids from normal skin expressed a 54 kDa caseinolytic activity (indicative of the latent form of MMP-1). There was no evidence of active MMP-1 in these culture fluids and no significant activity in the higher molecular weight regions of the gel. Representative zymograms from one basal cell carcinoma and one normal skin sample are shown in Figure 2A. Quantitative data from the entire group of specimens is shown in Figure 3.

To determine if caseinolytic activity could be detected in tissue extracts prepared from tissue immediately after surgery, four basal cell carcinoma specimens were frozen in liquid nitrogen upon arrival in the laboratory. Extracts prepared from these specimens were assayed for casein hydrolytic activity by zymography. In all four specimens, 54 kDa and 45 kDa caseinolytic activities were present (not shown). Thus, activity was not only detectable in the short-term organ culture fluids but was also measurable in the tumour tissue at the time of surgery.

Culture fluids from ten basal cell carcinomas and five normal skin specimens were examined by Western blotting for reactivity with antibodies to MMP-1, MMP-8 and MMP-13. Basal cell carcinoma reactivity with anti-MMP-1 antibody was observed in the 54 and 45 kDa regions of the gel, consistent with latent and active forms of MMP-1. Reactivity with anti-MMP-8 was seen as a band at approximately 50 kDa, while anti-MMP-13 reactivity was observed at 60 kDa. Organ culture fluids from normal skin showed anti-MMP-1 reactivity in the 54 kDa region (but not in the 45 kDa region). Reactivity with anti-MMP-8 was observed at approximately 50 kDa, while no reactivity with anti-MMP-13 was observed. Western blots from one basal cell carcinoma and one normal skin sample are shown in Figure 2B, along with blots from normal diploid fibroblasts and peripheral blood neutrophils (used as controls).

### Localization of MMP-1, MMP-8 and MMP-13 in histological section

Tissue sections were prepared from ten basal cell carcinoma specimens and stained with antibodies to MMP-1, MMP-8 and MMP-13. Staining with anti-MMP-1 was observed in all specimens. Intense anti-MMP-1 reactivity was observed throughout the stroma and in both the normal and tumour epithelium (Figure 4A). Anti-MMP-8 staining was also seen in tumour specimens. Staining was observed throughout the dermis; there was no staining in either the normal or tumour epithelium (Figure 4B). Anti-MMP-13 staining was also observed in the majority of specimens. Weak staining was observed in both the stroma and tumour epithelium, while more staining was observed in the normal epithelium adjacent to the tumour (Figure 4C).

In parallel, four normal skin specimens were stained with the same antibodies. As expected based on zymographic and Western blotting results, there was detectable (albeit, weak) staining with

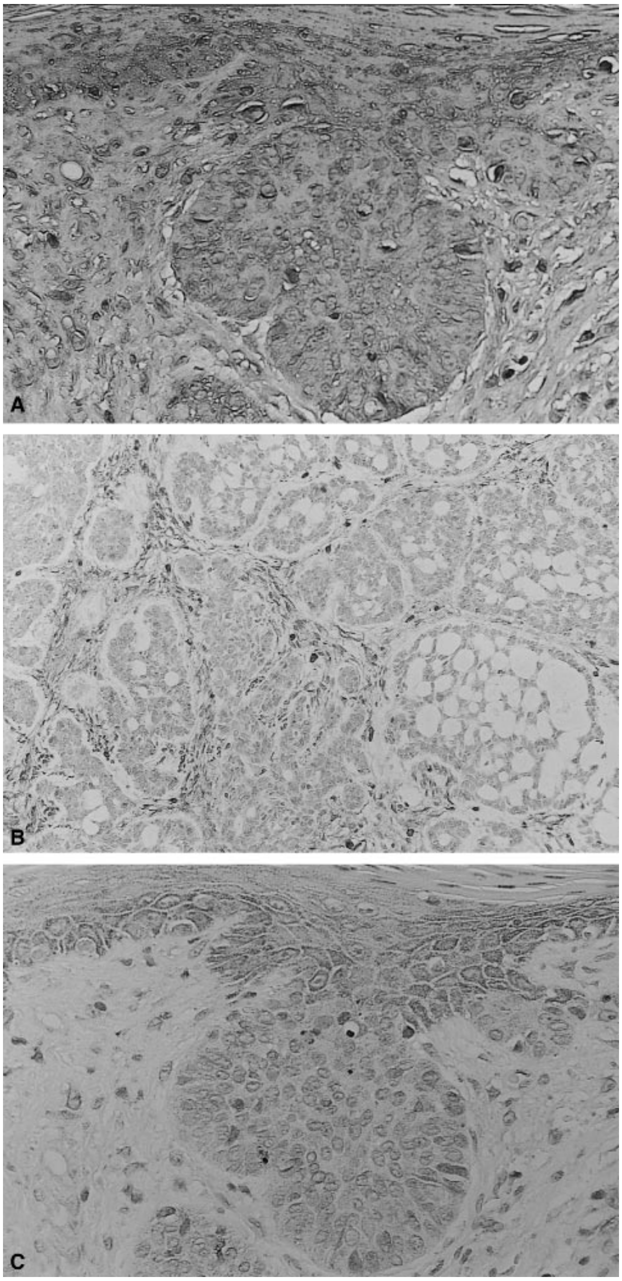

**Figure 4** Immunohistochemical detection of (**A**) MMP-1, (**B**) MMP-8 and (**C**) MMP-13 in representative sections from a basal cell carcinoma

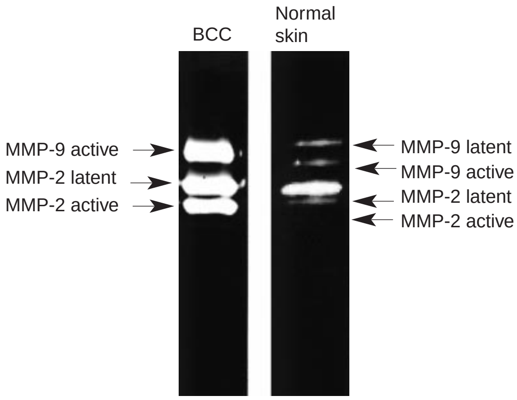

**Figure 5** Gelatinolytic activity in culture fluids from a representative basal cell carcinoma specimen and from a normal skin specimen

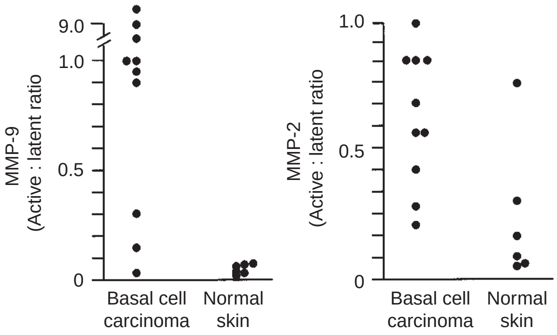

**Figure 6** Dot plot comparing ratios of active to latent MMP-9 and MMP-2 bands based on densitometry scanning of gelatin zymograms. Each dot is representative of an individual tumour or normal tissue specimen anti-MMP-1 in both the epidermis and dermis. Weak reactivity with anti-MMP-8 was also observed in the dermis while reactivity with anti-MMP-13 was not detectable (not shown).

### Gelatinase activity in basal cell carcinomas and normal skin

Organ culture fluids from the same specimens used for casein zymography were also examined for gelatinolytic activity by gelatin zymography. High levels of activity corresponding to both MMP-2 and MMP-9 were observed in all of the tumour specimens. In most of the culture fluids, a mixture of latent and active forms of both enzymes was observed. In certain of the culture fluids, only active forms of MMP-9 (e.g. 83–67 kDa) were present. Culture fluids from normal tissue specimens were examined in parallel. Consistent with past reports (Varani et al, 1995; Zeigler et al, 1996*b*), MMP-2 (72-kDa activity) was detected by gelatin zymography in these specimens, but MMP-9 (92-kDa activity) was undetectable or barely so. Latent forms of both enzymes predominated. Figure 5 shows gelatin zymograms from one basal cell carcinoma and one normal skin specimen. Quantitative data are shown in Figure 6. In additional studies (not shown) it was found that incorporation of 10 mM EDTA in the overnight washing buffer completely suppressed gelatinolytic activity while 2 mM PMSF was without effect. Western blotting demonstrated that the culture fluids contained appropriately-sized moieties reactive with antibodies to MMP-2 and MMP-9 (not shown).

Basal cell carcinoma specimens (ten in total) were also stained with antibodies to MMP-2 and MMP-9. Reactivity with the MMP-2 antibody was seen throughout the stroma as well as in both the normal and malignant epithelium in all of the specimens. Anti-MMP-9 staining was observed in six of the ten specimens. Most of the staining was seen in the normal epithelial cells immediately adjacent to the tumour. There was less stromal staining and very little staining in the tumour epithelium itself (not shown).

© 2000 Cancer Research Campaign 

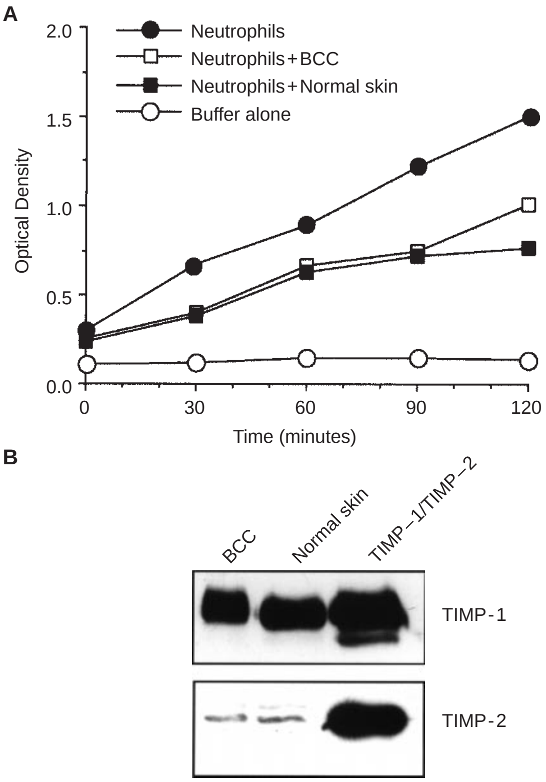

**Figure 7** (**A**) MMP inhibitor activity in culture fluids from basal cell carcinomas and normal skin. Values shown represent means values based on $n = 8$ tumour specimens and $n = 12$ normal tissue specimens. A standard curve was generated with TIMP-1 in each experiment, and the values were determined directly from the standard curve (see Table 1). (**B**) Western blotting of a representative basal cell carcinoma and a representative normal skin specimen with antibodies to TIMP-1 and TIMP-2

These immunohistochemical findings are consistent with previous observations by others (Pyke et al, 1992; Karelina et al, 1993; Kobayashi et al, 1996).

### Elaboration of MMP inhibitors by basal cell carcinomas and normal skin

Organ culture fluids from eight basal cell carcinomas were analysed for MMP inhibitor activity and for the presence of anti-TIMP-1- and anti-TIMP-2-reactive proteins. Organ culture fluids from 12 normal skin specimens were analysed for comparison. Similar levels of inhibitor activity were detected in all of the culture fluids (Figure 7A and Table 1). Based on Western blotting, MMP inhibitor activity in both the tumour culture and normal skin culture fluids appeared to be due almost entirely to TIMP-1 (Figure 7B).

**Table 1** Comparison of MMP inhibitor activity in organ culture fluids from basal cell carcinomas and normal skin

| Treatment | Percent inhibition of neutrophil MMP activity | TIMP-1 equivalents (ng 100 μl$^{-1}$) |
|---|---|---|
| TIMP-1 | | |
| 300 ng | 67 ± 8 | |
| 30 ng | 25 ± 4 | |
| 3 ng | 15 ± 3 | |
| 0.3 ng | 8 ± 3 | |
| Basal cell carcinoma | 35 ± 15 | 60 |
| Normal skin | 32 ± 6 | 58 |

MMP inhibition was assessed as described in the Materials and Methods section and in our recent report (Chi et al, 1999). Values shown are means and standard errors based on $n = 8$ for basal cell carcinomas and $n = 12$ for normal skin. TIMP-1 equivalent values were obtained by direct comparison with a TIMP-1 standard curve.

### DISCUSSION

The findings presented here are of interest from several standpoints. First is the identification of three different collagenolytic enzymes present in the tumour tissue. Previous studies have demonstrated high levels of collagenase activity associated with basal cell carcinomas (Goslen and Bauer, 1986; Bauer et al, 1997). Immunohistochemical techniques and in situ hybridization have demonstrated that epithelial tumours of skin (as well as epithelial tumours from other sites) elaborate detectable amounts of both MMP-1 and MMP-13 (Muller et al, 1991; Gray et al, 1992; Airola et al, 1997; Uria et al, 1997). Our results (e.g. Western blotting and immunohistology) indicate that MMP-8 is also represented in the tumour tissue. More importantly, however, our data (e.g. casein zymography in conjunction with Western blotting and immunohistology) suggest that although three different collagenolytic enzymes are detectable, the majority of the activity in the tumour tissue is reflective of MMP-1.

These studies are also of interest in regard to enzyme localization. Previous studies have shown that both the stroma and epithelium are capable of elaborating enzymes with collagenolytic activity under a variety of normal or pathological conditions (Muller et al, 1991; Polette et al, 1991; Shima et al, 1992; Gray et al, 1992; Airola et al, 1997; Johansson et al, 1997; Uria et al, 1997). In our basal cell carcinoma specimens, MMP-1 was highly expressed in the stroma surrounding the carcinoma as well as in both the normal and malignant epithelial cells. In contrast, MMP-13 was expressed in the normal epithelium adjacent to the tumour, but barely detectable in the tumour itself or in the tumour-associated stroma. MMP-8, on the other hand, was confined entirely to the stroma. With regard to gelatinolytic enzymes, MMP-9 was, like MMP-13, most highly expressed in the normal epithelial cells associated with the tumour rather than in the tumour cells or the surrounding stroma. On the other hand, MMP-2 was expressed throughout the stroma as well as in both the normal and malignant epithelium. These observations on distribution of the gelatinases are consistent with previous findings of others (Levy et al, 1991; Pyke et al, 1992; Shima et al, 1992; Karelina et al, 1993; Kobayashi et al, 1996). The implication of these findings is that there are multiple cellular sources for the collagenolytic and gelatinolytic MMPs detected in the tumour tissue. Past studies have focused on interactions between tumour cells and the surrounding stroma in regard to enzyme expression (Saarialho-Kere et al, 1993; Uria et al, 1997). Based on the present work, normal epithelial cells adjacent to the tumour and even circulating or tissue-infiltrating inflammatory cells should be considered as well.

Whether the cells expressing these various MMPs in the tissue are responsible for their synthesis is not clear. Our presumption is

 *© 2000 Cancer Research Campaign*

that resident cells in the epidermis and stroma are responsible for elaborating most of the MMPs (specifically, MMP-1, MMP-2, MMP-9 and MMP-13) detected in the skin. The source of MMP-8 is more problematic. MMP-8 was detected only in the stroma, where it was diffusely present throughout. Neutrophils are a major source of this enzyme (Grams et al, 1995), and it is reasonable to suggest that circulating neutrophils present in the tissue at the time of biopsy might be responsible for this enzyme. Alternatively, recent reports have indicated that in pathological conditions such as rheumatoid arthritis, chondrocytes, synovial fibroblasts and vascular endothelial cells elaborate MMP-8 mRNA and protein (Chubinskaya et al, 1996; Hanemaaijer et al, 1997). Of interest, the higher molecular weight forms associated with neutrophil MMP-8 (e.g. 68 kDa latent/58 kDa active) were not seen in the rheumatoid tissue. Rather, a form of approximately 50-kDa in size was the predominant species (Hanemaaijer et al, 1997). Since a similar-sized moiety was identified in culture fluids from the basal cell carcinomas (as well as in normal skin specimens), in situ biosynthesis in the skin is possible. Arguing against this, however, is the fact that we detected no MMP-8 reactivity by Western blotting in culture fluids from either dermal fibroblasts or dermal microvascular endothelial cells grown in monolayer culture (Figure 2). Under the same conditions, MMP-1 and MMP-13 reactivity was detected. Furthermore, it was observed by Hanemaaijer et al (1997) and by us (unpublished observation) that a 50 kDa fragment could also be detected in neutrophil releasate by Western blotting. In our studies there was no detectable caseinolytic activity associated with this moiety. Based on these considerations, we favour the hypothesis that neutrophils present in the blood or tissue at the time of surgery constitute the major source of the stromal MMP-8 reactivity and that the 50-kDa species represents a stable – but enzymatically not very active – form of the protein. This does not rule out contributions by resident cells.

Perhaps the most interesting aspect of the present study is the relationship between enzyme/inhibitor profile and specimen type. The profile of enzymes and inhibitors in the tumour specimens was clearly different from the pattern expressed in normal human skin maintained under the same serum-free, growth factor-free conditions (present study and Varani et al, 1995; Zeigler et al, 1996*b*; Chi et al, 1999). Specifically, the tumour specimens expressed high levels of MMP-1 and MMP-9 (with active forms of both enzymes predominating), while lower levels of the same two enzymes (primarily latent forms) were expressed in normal skin. Additional distinguishing features included the presence of detectable MMP-13 reactivity (by Western blotting and immunostaining) in the tumour specimens but a lack of reactivity associated with the normal skin, and the presence of active MMP-2 in the tumour specimens while much of the MMP-2 in the normal skin specimens was in the latent form. On the other hand, the tumour specimens and normal tissue specimens demonstrated comparable MMP-8 reactivity by Western blotting and expressed similar levels of MMP inhibitor activity (reflective of the presence of TIMP-1 but not TIMP-2 in both cases). The high level of MMP activity in tumour tissue as compared to that seen in normal skin (without a corresponding change in MMP inhibitor activity) is consistent with the hypothesis that these enzymes contribute to tissue destruction and local tumour invasion in basal cell carcinoma.

Although differences in MMP expression between basal cell carcinomas and normal skin support a role for these enzymes in local invasion by basal cell tumours, these findings do not prove ‘cause and effect’. Of interest in this regard, however, we have previously reported that the MMP profile in normal skin is altered when the tissue is exposed to exogenous growth factors in organ culture growth medium (Varani et al, 1995; Zeigler et al, 1996*b*; Chi et al, 1999). Under the influence of exogenous growth factors, there is up-regulation and activation of MMP-1 and MMP-9, concomitant with activation of MMP-2, but without a change in expression of TIMP-1 or TIMP-2. Thus, the pattern of MMP/MMP inhibitor expression is altered in growth factor-treated normal skin so as to resemble the profile expressed endogenously by the malignant tumours. Concomitantly it has been shown that the same growth factors that up-regulate and activate MMPs in normal skin also promote stromal invasion by the epithelium in this tissue (Fligiel and Varani, 1993; Zeigler et al, 1996*a*). Stromal invasion in growth factor-treated skin is inhibitable with the MMP inhibitor, TIMP-2 (Zeigler et al, 1996*b*). Taken together with these previously published data, the present findings indicate that MMP up-regulation and activation without a concomitant change in MMP inhibitor expression is associated with local tumour invasion in both the experimental invasion model and in actual malignant tumours of skin. Of interest, the ability to maintain tumour tissue in organ culture should allow us to directly assess whether treatments which interfere with MMP induction (Zeigler et al, 1999) or function (Zeigler et al, 1996*b*) modulate the histopathological changes that occur during culture. A major advantage of organ culture is that while it allows for maintenance of tissue under ‘in situ’ conditions, it is, in fact, an in vitro system, and can be manipulated as readily as other in vitro culture systems.

Although there was a large difference between normal skin and basal cell carcinomas in enzyme/inhibitor profile, we were unable to clearly distinguish tumours defined as aggressive growth pattern basal cell carcinoma from tumours with less aggressive growth patterns on the basis of enzyme/inhibitor profile. Clinical studies have demonstrated that tumours identified as aggressive growth pattern types tend to invade more deeply, and have a higher recurrence rate (perhaps not surprisingly) than nodular or superficial basal cell tumours (Salasche and Amonette, 1981; Lang and Maize, 1986). Additionally, the more aggressive tumours tend to be surrounded by a discontinuous basement membrane, in contrast to nodular or superficial types of basal cell carcinomas, which often have an intact basement membrane (Johnson et al, 1993). The question arises then that if the collagenases and gelatinases studied here are not responsible for the differences in aggressiveness among various basal cell carcinomas, what is. There are a number of possibilities. Aggressive growth pattern may reflect differences in levels of MMPs other than those examined here. In regard to this, we previously demonstrated no differences in stromelysin expression between aggressive growth pattern basal cell carcinomas and the more indolent types (Majmudar et al, 1994), while differences between normal skin and basal cell tumours of all types was large. Another possibility is that tumour aggressiveness may reflect differences in matrix synthesis rather than breakdown. Reduced matrix production is a hallmark of many types of tumours, although squamous epithelial cell tumours may not follow this pattern (Varani et al, 1991). Another possibility is that aggressiveness may reflect characteristics of the host rather than the tumour. Additional studies will be needed to address these various possibilities.

In closing, it should be pointed out that while MMP up-regulation and activation is a consistent finding in basal cell carcinomas,

© 2000 Cancer Research Campaign it is not unique to skin tumours. Quite the opposite; enhanced MMP production occurs in other skin conditions where homeostasis is upset (Kahari and Saarialho-Kere, 1997). Our own studies have shown that MMP-1 and MMP-9 are induced in two other conditions of the skin – i.e. in psoriasis (Varani et al, 1998) and after acute exposure to ultraviolet light (Fisher et al, 1996, 1997). In both conditions, damage to the extracellular matrix occurs but in neither is stromal invasion by epithelial cells a prominent feature. Thus, MMP expression is strongly correlated with damage to the extracellular matrix rather than with invasion per se.

### ACKNOWLEDGEMENT

This study was supported in part by grant CA 60958 from the USPHS.

### REFERENCES

Airola K, Kariniemi A-L, Kahari V-M and Saarialho-Kere U (1997) Human collagenase-3 (MMP-13) is expressed in malignant squamous epithelia of the skin. *J Invest Dermatol* **109**: 225–231
Bauer EA, Gordon JM, Reddick ME and Eisen AZ (1977) Quantitation and immunochemical localization of human skin collagenase in basal cell carcinoma. *J Invest Dermatol* **69**: 363–367
Chi Y, Zeigler ME, Walker J, Perone P and Varani J (1999) Elaboration of matrix metalloproteinase inhibitors by human skin in organ culture and by skin cells in monolayer culture: relationship to invasion. *Invasion Metastasis* **18**: 27–34
Chubinskaya S, Huch K, Mikecz K, Cs-Zabo G, Hasty KA, Kuettner KE and Cole AA (1996) Chondrocyte MMP-8: upregulation of neutrophil collagenase by interleukin 1β in human cartilage from knee and ankle joints. *Lab Invest* **74**: 232–240
Coussens LM and Werb Z (1996) Matrix metalloproteinase expression and neoplasia. *Chem Bio* **3**: 895–904
Erkell LJ and Schirrmacher V (1988) Quantitative in vitro assay for tumor cell invasion through extracellular matrix or into protein gels. *Cancer Res* **48**: 6933–6937
Fisher GJ, Datta SC, Talwar HS, Wang Z-Q, Varani J, Kang S and Voorhees JJ (1996) Molecular basis of sun-induced premature skin ageing and retinoid antagonism. *Nature (Lond)* **379**: 335–339
Fisher GJ, Wang ZQ, Datta SC, Varani J, Kang S and Voorhees JJ (1997) Pathophysiology of premature skin aging induced by ultraviolet light. *New Engl J Med* **337**: 1419–1428
Fligiel SEG and Varani J (1993) In situ epithelial cell invasion in organ culture. *Invasion Metastasis* **13**: 225–233
Galis ZS, Sukhova GK, Lark MW and Libby P (1994) Increased expression of matrix metalloproteinases and matrix degrading activity in vulnerable regions of human atherosclerotic plaques. *J Clin Invest* **94**: 2493–2503
Gibbs DF, Shanley TP, Warner RO, Murphy HS, Varani J and Johnson KJ (1999) Role of matrix metalloproteinases in models of macrophage-dependent acute lung injury. *Am J Respir Cell Mol Biol* **20**: 1145–1154
Goslen JB and Bauer E (1986) Basal cell carcinoma and collagenase. *J Dermatol Surg Oncol* **12**: 812–817
Grams F, Crimmin M, Hinnes L, Huxley P, Pieper M, Tschesche H and Bode W (1995) Structural determination and analysis of human neutrophil collagenase. *Biochemistry* **34**: 14012–14020
Gray ST, Wilkins RJ and Yun K (1992) Interstitial collagenase gene expression in oral squamous cell carcinoma. *Am J Pathol* **141**: 301–306
Hanemaaijer R, Soras T, Konttinen YT, Ding Y, Sutinen M, vesser H, van Hinsbergh VW, Helaakoski T, Kainulainen T, Ponka H, Tschesche H and Salo T (1997) Matrix metalloproteinase-8 is expressed in rheumatoid synovial fibroblasts and endothelial cells. *J Biol Chem* **272**: 31504–31509
Hasty KA, Rife RA, Kang AH and Stuart JM (1990) The role of stromelysin in the cartilage destruction that accompanies inflammatory arthritis. *Arthritis Rheum* **33**: 388–397
Hu C-L, Crombie G and Franzblau C (1978) A new assay for collagenolytic activity. *Analyt Biochem* **88**: 638–643
Huber AR, Ellis S, Johnson KJ, Dixit VM and Varani J (1992) Monocyte diapedesis through an in vitro vessel wall construct: Inhibition with monoclonal antibodies to thrombospondin. *J Leuk Biol* **52**: 524–528
Inoue M, Kratz G, Haegerstrand A and Stahle-Backdahl M (1995) Collagenase expression is rapidly induced in wound-edge keratinocytes after acute injury in human skin, persists during healing, and stops at re-epithelialization. *J Invest Dermatol* **104**: 479–483
Johansson N, Airola K, Grenman R, Kariniemi A-L, Saarialho-Kere U and Kahari V-M (1997) Expression of collagenase-3 (matrix metalloproteinase-13) in squamous cell carcinomas of the head and neck. *Am J Pathol* **151**: 499–508
Johnson TM, Nelson BR, Jensen T and Majmudar G (1993) Matrix metalloproteinases in local tumor invasion in non-melanoma skin cancer. *Cancer Bull* **45**: 238–244
Kahari VM and Saarialho-Kere (1997) Matrix metalloproteinases in the skin. *Exp Dermatol* **6**: 199–213
Karelina T, Hruza GJ, Goldberg GI and Eisen AZ (1993) Localization of 92-kDa type IV collagenase in human skin tumors: comparison with normal human fetal and adult skin. *J Invest Dermatol* **100**: 159–165
Kobayashi T, Onoda N, Takagi T, Hori H, Hattori S, Nagai Y, Tajima S and Nishikawa T (1996) Immunolocalization of human gelatinase (type IV collagenase, MMP-9) and TIMP in normal epidermis and some epidermal tumors. *Arch Dermatol Res* **288**: 239–244
Kramer RH and Nicolson GL (1979) Interaction of tumor cells with vascular endothelial cell monolayers: A model for metastatic invasion. *Proc Natl Acad Sci USA* **76**: 5704–5708
Lang PG and Maize JC (1986) Histologic evolution of recurrent basal cell carcinoma and treatment implications. *J Am Acad Dermatol* **14**: 186–196
Levy AT, Cioce V, Sobel ME, Barbisa S, Grigioni WF, Liotta LA and Stetler-Stevenson WB (1991) Increased expression of the $M_r$ 72 000 type IV collagenase in human colonic adenocarcinoma. *Cancer Res* **51**: 439–444
Majmudar G, Nelson BR, Jensen TC, Voorhees JJ and Johnson TM (1994) Increased expression of stromelysin-3 in basal cell carcinomas. *Mol Carcinogen* **9**: 17–23
Mareel MM, Kint J and Meyvisch C (1979) Methods of study of the invasion of malignant C3H-mouse fibroblasts into embryonic chick heart in vitro. *Virchows Arch B: Cell Pathol* **30**: 95–111
Muller D, Breathnach R, Englelmann A, Millon R, Bronner G, Flesch H, Dumont P, Eber M and Abecassis J (1991) Expression of collagenase-related metalloproteinase genes in human lung or head and neck tumours. *Int J Cancer* **48**: 550–556
Mulligan MS, Desrochers PE, Chinnaiyan AM, Gibbs DF, Varani J, Johnson KJ and Weiss SJ (1993) In vivo suppression of immune complex-induced alveolitis by secretory leukoproteinase inhibitor and tissue inhibitor of metalloproteinases-2. *Proc Natl Acad Sci USA* **90**: 11523–11527
Polette M, Clavel C, Muller D, Abecassis J, Binninger I and Birembaut P (1991) Detection of mRNAs encoding collagenase I and stromelyin 2 in carcinomas of the head and neck by in situ hybridization. *Invasion Metastasis* **11**: 76–83
Pyke C, Ralfiaer E, Huhtala P, Hurskainen T, Dano K and Tryggvason K (1992) Localization of messenger RNA for $M_r$ 72 000 and 92 000 type IV collagenases in human skin cancers by in situ hybridization. *Cancer Res* **52**: 1336–1341
Reich R, Thompson EW, Iwamoto Y, Martin GR, Deason JR, Fuller GC and Miskin R (1988) Effects of inhibitors of plasminogen activators, serine proteinases and collagenase IV on the invasion of basement membranes by metastatic cells. *Cancer Res* **48**: 3307–3312
Rosenthal EL, Johnson TM, Allen ED, Apel IJ, Punturieri A and Weiss SJ (1998) Role of plasminogen activator and matrix metalloproteinase systems in epidermal growth factor- and scatter factor-stimulated invasion of carcinoma cells. *Cancer Res* **58**: 5221–5230
Saarialho-Kere UK, Kovacs SO, Pentland AP, Olerud JE, Welgus HG and Parks WC (1993) Cell–matrix interactions modulate interstitial collagenase expression by human keratinocytes actively involved in wound healing. *J Clin Invest* **92**: 2858–2866
Salasche SJ and Amonette RA (1981) Morpheaform basal cell epitheliomas. *J Dermatol Surg Oncol* **7**: 387–394
Shapiro SD, Fliszar CJ, Broekelmann TJ, Mecham RP, Senior RM and Welgus HG (1995) Activation of the 92-kDa gelatinase by stromelysin and 4-aminophenylmercuric acetate. *J Biol Chem* **270**: 6351–6356
Shima I, Sasaguri Y, Kusukawa J, Yamana H, Fujita H, Kakegawa T and Morimatsu M (1992) Production of matrix metalloproteinase-2 and metalloproteinase-3 related to malignant behavior of esophageal carcinoma: a clinico-pathologic study. *Cancer* **70**: 2747–2753
Sottrup-Jensen L and Birkedal-Hansen H (1989) Human fibroblast collagenase–α-macroglobulin interactions. *J Biol Chem* **264**: 393–401
Springman EB, Angleton EL, Birkedal-Hansen H and Van Wart H (1990) Multiple modes of activation of latent human fibroblast collagenase: evidence for the role of a Cys 73 active-site zinc complex in latency and a cysteine switch mechanism for activation. *Proc Natl Acad Sci USA* **87**: 364–368

© 2000 Cancer Research Campaign

Sympson CJ, Talhouk RS, Alexander CM, Chin JR, Clift SM, Bissell MJ and Werb Z (1994) Targeted expression of stromelysin-1 in mammary gland provides evidence for a role of proteinases in branching morphogenesis and the requirement for an intact basement membrane for tissue-specific gene expression. *J Cell Biol* **125**: 681–693

Uria JA, Stahle-Backdahl M, Seiki M, Fueyo A and Lopez-Otin C (1997) Regulation of collagenase-3 expression in human breast carcinoma is mediated by stromal–epithelial cell interactions. *Cancer Res* **57**: 4882–4888

Varani J, Fligiel SEG, Till GO, Kunkel RG, Ryan US and Ward PA (1985) Pulmonary endothelial cell killing by human neutrophils: possible involvement of hydroxyl radical. *Lab Invest* **53**: 656–663

Varani J, Schuger L, Fligiel SEG, Inman DR and Chakrabarty S (1991) Production of fibronectin by human tumor cells and interaction with exogenous fibronectin: comparison of cell lines obtained from colon adenocarcinomas and squamous carcinomas of the upper aerodigestive tract. *Int J Cancer* **47**: 421–425

Varani J, Fligiel SEG, Schuger L, Perone P, Inman DR, Griffiths CEM and Voorhees JJ (1993*a*) Effects of all-trans retinoic acid and $Ca^{2+}$ on human skin in organ culture. *Am J Pathol* **142**: 189–198

Varani J, Larson BK, Perone P, Inman DR, Fligiel SEG and Voorhees JJ (1993*b*) All-trans retinoic acid and $Ca^{2+}$ differentially influence extracellular matrix production by human skin in organ culture. *Am J Pathol* **142**: 1813–1822

Varani J, Perone P, Griffiths CEM, Inman DR, Fligiel SEG and Voorhees JJ (1994) All-trans retinoic acid (RA) stimulates events in organ-cultured human skin that underlie repair. *J Clin Invest* **94**: 1747–1756

Varani J, Perone P, Inman DR, Burmeister W, Scholenberger SB, Fligiel SEG, Sitrin RG and Johnson KJ (1995) Human skin in organ culture: elaboration of proteolytic enzymes in the presence and absence of exogenous growth factors. *Am J Pathol* **146**: 210–217

Varani J, Zeigler M, Perone P, Carey TE and Datta SC (1997) Human squamous carcinoma cell invasion in organ-cultured skin. *Cancer Lett* **111**: 51–57

Varani J, Kang S, Stoll S and Elder JT (1998) Human psoriatic skin in organ culture: comparison with normal skin exposed to exogenous growth factors and effects of an antibody to the EGF receptor. *Pathobiology* **66**: 253–259

Zeigler ME, Krause S, Karmiol S and Varani J (1996*a*) Growth factor-induced epidermal invasion of the dermis in human skin organ culture: dermal invasion correlated with epithelial cell motility. *Invasion Metastasis* **16**: 3–10

Zeigler ME, Dutcheshen NT, Gibbs DFG and Varani J (1996*b*) Growth factor-induced epidermal invasion of the dermis in human skin organ culture: expression and role of matrix metalloproteinases. *Invasion Metastasis* **16**: 11–18

Zeigler ME, Chi Y, Schmidt T and Varani J (1999) Role of ERK and JNK pathways in regulating cell motility and matrix metalloproteinase 9 production in growth factor-stimulated human epidermal keratinocytes. *J Cell Physiol* **180**: 271–284

© 2000 Cancer Research Campaign
